# Adherence to the Mediterranean Diet and Dietary Potassium Intake: A Narrative Review of Epidemiological Evidence

**DOI:** 10.3390/nu18040551

**Published:** 2026-02-07

**Authors:** Lanfranco D’Elia, Saverio Stranges, Francesco P. Cappuccio, Pasquale Strazzullo, Ferruccio Galletti

**Affiliations:** 1 Department of Clinical Medicine and Surgery, “Federico II” University of Naples Medical School, 80131 Naples, Italy; saverio.stranges@uwo.ca (S.S.); galletti@unina.it (F.G.); 2 “Federico II” AOU of Naples, Via Sergio Pansini 5, 80131 Naples, Italy; 3 Department of Epidemiology & Biostatistics, Schulich School of Medicine and Dentistry, Western University, London, ON N6G 2M1, Canada; 4 Department of Family Medicine, Schulich School of Medicine and Dentistry, Western University, London, ON N6G 2M1, Canada; 5 Department of Medicine, Schulich School of Medicine and Dentistry, Western University, London, ON N6A 5A5, Canada; 6 Warwick Applied Health, Warwick Medical School, University of Warwick, Coventry CV4 7AL, UK; f.p.cappuccio@warwick.ac.uk; 7 University Hospitals Coventry & Warwickshire NHS Trust, Coventry CV4 7AL, UK; 8 Internal Medicine, University of Naples Federico II, 80131 Naples, Italy; strazzul@unina.it

**Keywords:** Mediterranean diet, potassium, sodium-to-potassium ratio, cardiovascular health, diet quality, nutritional epidemiology, Mediterranean dietary pattern

## Abstract

Background. The Mediterranean dietary pattern (MDP) is recognised as one of the most evidence-based dietary models for the prevention of non-communicable diseases (NCDs). Its plant-rich composition suggests an inherently high potassium intake, yet epidemiological findings on the association between MDP adherence and potassium intake remain heterogeneous. The present review aims to summarise and critically evaluate the available evidence on the association between adherence to the MDP and dietary potassium intake in the adult population. Methods. We conducted a narrative review of observational, longitudinal and interventional studies evaluating the relationship between MDP adherence and dietary potassium intake (self-reported assessment and/or 24 h urinary potassium). MEDLINE/PubMed was searched from inception to 30 October 2025, additional studies were identified by reference screening. Results. From a total of 263 studies retrieved, 10 eligible studies (7 cross-sectional, 1 longitudinal, 2 randomised controlled trials) from Europe, Asia and North America were synthesised. Questionnaire-based studies consistently indicated higher potassium intake with greater MDP adherence, whereas biomarker-based findings were more variable and often attenuated, particularly in studies relying on single or unvalidated 24 h urine collections and selected samples. Overall risk of bias was high for most observational studies, while randomised trials were generally rated as having some concerns. Conclusions. Higher MDP adherence is generally associated with higher potassium intake, but estimates vary by how MDP adherence is defined and scored, the potassium assessment method, and population context. Current evidence remains insufficient to quantify potassium’s potential contribution as a candidate mediator without formal mediation analyses and robust exposure assessment, including repeated validated 24 h urine collections. Standardised scoring, routine reporting of potassium, sodium, and the Na/K ratio, and triangulation across dietary, biomarker and intervention evidence are key priorities to strengthen inference.

## 1. Introduction

Dietary habits are among the most powerful determinants of human health, strongly influencing metabolic balance, inflammation, and longevity. Over recent decades, increasing evidence has demonstrated that overall diet quality plays a crucial role in shaping the risk and progression of non-communicable diseases (NCDs), including metabolic, cardiovascular [1,2], and neurodegenerative disorders [3]. Among dietary regimens, the Mediterranean dietary pattern (MDP) is widely regarded as a leading model of optimal nutrition, supported by extensive evidence linking its adoption to improved cardiometabolic and health outcomes [4,5]. Defined by a high intake of plant-based foods, namely fruits, vegetables, legumes, nuts, and whole grains, along with olive oil as the principal source of fat, moderate consumption of fish, a low intake of red and processed meats, and a cautious and limited intake of alcoholic beverages (preferably wine with meals), the MDP provides a balanced and nutrient-dense dietary structure [6]. In addition to its well-documented health advantages, the MDP has been recognised as one of the dietary patterns most closely aligned with the planetary-health diet proposed by the EAT-Lancet Commission, underscoring its relevance as a sustainable nutritional model for the Anthropocene [7].

The health-promoting effects of the MDP derive from the synergistic interactions among its bioactive components, including monounsaturated fats, polyphenols, fibre, and essential micronutrients, which collectively modulate oxidative stress, inflammation, and vascular and metabolic function [8]. Among these micronutrients, potassium plays a particularly relevant role [9]. As the major intracellular cation, potassium is essential for maintaining vascular tone, neuromuscular function, and electrolyte homeostasis. Adequate potassium intake has consistently been associated with improved blood pressure regulation [10], a reduced risk of stroke [11] and type 2 diabetes [12], and enhanced endothelial function [13].

Unlike sodium, which is widely present in processed and refined foods, potassium is naturally abundant in fresh, unprocessed plant-based foods [6]. Consequently, dietary patterns rich in fruits, vegetables, and legumes, such as the MDP, inherently support higher potassium intake. Notably, potassium cannot be meaningfully discussed without considering sodium, as the two operate in a tightly interconnected physiological balance. It is the Na/K ratio, rather than the absolute intake of either electrolyte alone, that more accurately reflects dietary quality and cardiovascular risk. Excess dietary sodium raises blood pressure and impairs vascular function, whereas potassium promotes natriuresis, supports endothelial health, and counteracts sodium’s adverse haemodynamic effects. By improving this crucial balance, the MDP’s nutrient profile may contribute to its metabolic and vascular benefits, helping to mitigate the negative consequences of excessive sodium consumption [10,14,15].

Nevertheless, despite the strong biological rationale linking potassium intake to MDP adherence, current findings are not entirely consistent. Evidence from observational and interventional studies reveals considerable heterogeneity related to differences in study populations, dietary assessment tools, and biomarkers [16,17,18,19,20,21,22,23,24,25]. Studies using self-reported methods such as food frequency questionnaires (FFQs) typically demonstrate stronger associations between MDP adherence and potassium intake, reflecting long-term dietary behaviours. In contrast, those relying on 24 h urinary potassium excretion (the gold-standard biomarker for intake assessment [26,27]) often yield attenuated or null results. These discrepancies reflect key methodological challenges, including variability in dietary assessment tools, limitations of single urine collections, and heterogeneity in regional adaptations of the MDP.

In addition, differences in national food composition databases, cultural variations in discretionary salt use, and the adoption of modified MDPs outside the Mediterranean region may further influence estimates of potassium and sodium–potassium balance. Contemporary adaptations of the MDP may retain its broad structure yet diverge substantially in nutrient composition, particularly with respect to minerals, leading to inconsistent findings across populations [15].

These inconsistencies highlight the need for a comprehensive synthesis of available evidence and a critical appraisal of methodological limitations. Accordingly, the present review aims to summarise and critically evaluate the available evidence on the association between MDP adherence and dietary potassium intake in the adult population, integrating findings from observational and interventional studies, and considering both self-reported assessments and biomarker-based measures.

## 2. Materials and Methods

A targeted literature search was conducted on MEDLINE/PubMed up to 30 October 2025. The search strategy was applied in this database without language or date restrictions, combining free-text Mediterranean diet terms searched in Title/Abstract with potassium terms captured using both controlled vocabulary (MeSH) and Title/Abstract keywords. Specifically, the search string was: (“Mediterranean diet” [Title/Abstract] OR “Mediterranean dietary pattern” [Title/Abstract]) AND (“Potassium” [MeSH] OR “potassium” [Title/Abstract]). In addition, we performed citation-based searching by screening the reference lists of recent reviews and of studies judged relevant at the full-text stage, and by iteratively checking references until no further eligible records were identified.

This manuscript is an interpretive narrative review aimed at summarising and critically appraising epidemiological and trial evidence linking MDP adherence with potassium-related outcomes. We used a structured search and PICOS (Population, Intervention/Exposure, Comparison, Outcomes, Study Design)-informed eligibility criteria to enhance transparency and to clearly define the scope of included evidence. We also applied formal risk-of-bias tools to support critical appraisal and interpretation within a narrative framework. Consistent with this design, we did not preregister a protocol, undertake multi-database retrieval, perform duplicate independent screening/data extraction, or conduct meta-analytic pooling; formal assessment of publication bias was therefore not undertaken.

Eligible studies were selected according to predefined PICOS criteria. Population: adults. Intervention/Exposure: Mediterranean diet or MDP, assessed through a priori/a posteriori dietary indices or adherence scores, or through a predefined Mediterranean-style dietary intervention. Comparator: higher versus lower MDP adherence categories and/or MDP intervention versus habitual diet/control. Outcomes: dietary potassium intake and/or urinary potassium excretion. Study design: observational (cross-sectional, case–control, cohort) and intervention studies (randomised controlled trials). Studies were excluded if they were conducted in non-human models, involved children/adolescents, were restricted to patient populations (for observational designs), did not evaluate MDP exposure, did not report potassium intake or urinary potassium measures, or were not original research articles (e.g., reviews, editorials, protocols). This restriction was applied to observational designs to reduce confounding related to clinical status and disease-driven dietary counselling. Randomised controlled trials conducted in clinical populations were retained to provide context-specific experimental evidence on the feasibility and short-term effects of MDP interventions on potassium-related outcomes.

As this was a descriptive narrative review, screening, study selection, and data extraction processes were conducted by a single author (LD). This approach may increase the risk of selection and extraction errors and subjective interpretation. It is therefore acknowledged as a limitation. The risk of bias in epidemiological studies was assessed using the Risk Of Bias In Non-randomised Studies of Exposures (ROBINS-E) tool [28]. Risk of bias in randomised controlled trials was assessed using the revised Cochrane risk-of-bias tool for randomised trials (RoB 2) [29]. For ROBINS-E, critical confounders were defined a priori as age, sex, total energy intake (where applicable), socioeconomic status/education, physical activity, BMI/adiposity, smoking, renal function, and diuretic use/antihypertensive medication (Table 1).

## 3. Results

From a total of 263 studies retrieved, 10 eligible studies were synthesised (Table 2): 7 cross-sectional analyses (5 questionnaire/recall-based and 2 based on 24 h urine), 1 longitudinal cohort analysis, and 2 randomised controlled trials.

**Figure 1:**
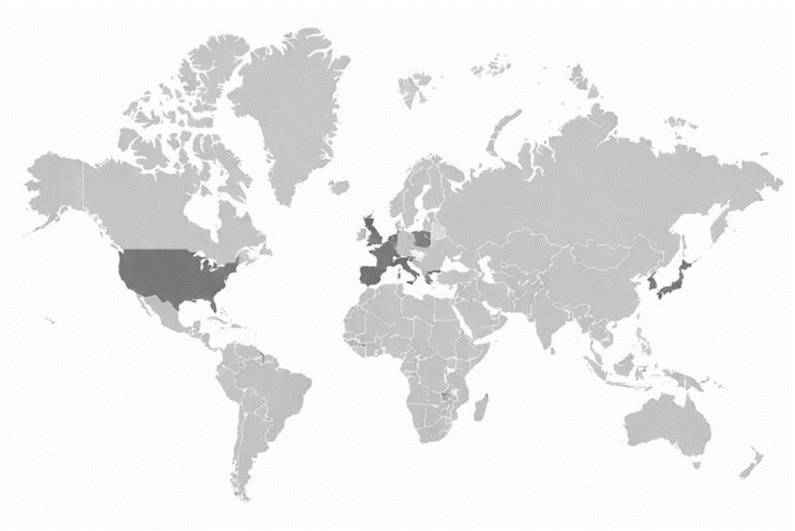
Geographic distribution of included studies. Countries shaded in dark grey indicate the locations of the 10 studies included in this review (Europe: Spain, France, Italy, Greece, Portugal, and the multicentre European NU-AGE trial; Asia: Japan and Korea; North America: USA). This figure summarises the geographic spread of the evidence base and is intended to support interpretation of heterogeneity across settings (e.g., food environments, sodium sources, and culinary practices relevant to potassium and Na/K). Shading reflects study setting(s) and does not represent study sample size, study quality, or population representativeness.

Across studies, samples included both men and women and ranged widely in size, from 50 participants in a pilot crossover trial to 17,197 in the largest population-based analysis. Na/K outcomes were reported in one study as dietary Na/K [20], and in two studies as urinary Na/K [19,21]. The evidence base was geographically diverse, with studies conducted predominantly in Europe (Spain, France, Italy, Greece, Portugal, and the multicentre NU-AGE setting), alongside Asia (Japan, Korea) and North America (USA), as summarised in Figure 1.

### 3.1. Epidemiological Evidence (Observational and Longitudinal)

A growing body of epidemiological research has examined the relationship between adherence to the MDP and potassium exposure across diverse populations, using both self-reported dietary assessments and urinary biomarkers [16,17,18,19,20,21,22,23]. Included studies differed in design, MDP score definitions, and potassium assessment approach (Table 2).

Findings are also described by MDP score definition (a posteriori pattern-derived scores vs. a priori indices/brief scores vs. intervention-specific indices). To facilitate comparability, findings are synthesised by potassium assessment method (questionnaire-/recall-based estimates vs. urinary biomarkers), and comparable effect descriptors are reported narratively where available (gradients across adherence categories, highest-versus-lowest differences, or regression coefficients). Urine collection characteristics are summarised in Table 3.

#### 3.1.1. Questionnaire/Recall-Based Potassium Assessment

A posteriori dietary pattern (FFQ-derived potassium).

Serra-Majem et al. analysed 17,197 Spanish university graduates and derived an MDP a posteriori using principal component analysis from a validated 136-item FFQ [16]. Potassium intake was estimated from FFQ data using Spanish food composition tables and energy-adjusted. Higher MDP adherence was associated with higher potassium intake, whereas a Western dietary pattern showed an inverse association. Effect metric: Potassium increased across MDP quintiles (3.7 to 6.1 g/day), while Western pattern adherence was associated with lower potassium (5.4 to 4.2 g/day). In the ROBINS-E appraisal (Table 1), the study was rated at overall high risk of bias, driven primarily by construct overlap/common-method structure (MDP adherence and potassium derived from the same FFQ-based framework) and limited representativeness. Higher MDP adherence was also associated with a lower prevalence of multiple micronutrient inadequacies, consistent with the broader nutrient adequacy of the pattern.

Score-based MDP indices with dietary instrument-based potassium (FFQ or diet-history tools).

Rumawas et al. applied an MDP-Style Score (0–100) derived from a validated 126-item FFQ in 3021 adults from the Framingham Offspring cohort [22]. Potassium intake increased with higher MDP adherence. Effect metric: Graded increase across quintiles (≈2.4 to 3.3 g/day). In ROBINS-E, overall high risk was driven primarily by common-method structure/construct overlap and lack of biomarker corroboration, with some additional concerns related to exposure measurement and participant selection (Table 1).

Kanauchi et al. evaluated a Japanese-adapted 13-component MDP score (0–4/5–7/8–13) in 1048 adults using the Brief-type Self-Administered Diet History Questionnaire (BDHQ), with potassium estimated from the same instrument using Japanese food composition tables [18]. Effect metric: Graded increase from low to high adherence (≈2.5 to 3.5 g/day). In ROBINS-E, overall high risk was driven primarily by outcome measurement limitations and construct overlap/common-method structure, with some additional concerns for confounding and participant selection (Table 1).

Malavolti et al. assessed MDP adherence using the Italian Mediterranean Index (IMI) and the Mediterranean–DASH Intervention for Neurodegenerative Delay (MIND) score in 719 Northern Italian adults; potassium and sodium were estimated from EPIC-FFQ data linked to mineral composition obtained by inductively coupled-plasma mass spectrometry for 908 commonly consumed foods [20]. Effect metric: Potassium increased (3.1 to 3.6 g/day) with stable sodium (2.2 vs. 2.1 g/day), consistent with improved dietary Na/K. In ROBINS-E, overall high risk was driven primarily by FFQ-based outcome measurement limitations and potential construct overlap/common-method structure, despite the methodological strength of direct chemical quantification of food mineral content (Table 1).

Score-based MDP index with recall-based potassium (24 h dietary recall).

Feart et al. studied 1595 older adults in France [17]. MDP adherence was assessed using a nine-point MDP score derived from a validated 148-item FFQ; potassium intake was estimated from a single 24 h dietary recall conducted by trained dietitians. Effect metric: Using MDP categories (0–3/4–5/6–9), 24 h recall potassium intake increased in both sexes (men: 2.9 to 3.0 to 3.1 g/day; *p* = 0.04; women: 2.4 to 2.5 to 2.7 g/day; *p* < 0.0001), consistent with higher potassium exposure at greater MDP adherence. In ROBINS-E, overall high risk was driven mainly by high risk in confounding and some concerns in outcome measurement (single 24 h recall), with other domains largely low-risk (Table 1).

Na/K reporting within questionnaire-based studies.

Within questionnaire-based studies, Na/K outcomes were reported less consistently. Where available, sodium was presented alongside potassium, enabling distinction between increased potassium intake and evidence consistent with improved electrolyte balance (dietary Na/K) [20].

#### 3.1.2. Urinary Biomarker-Based Potassium Assessment (24 h Urine)

Studies using urinary biomarkers have produced less consistent findings, and key protocol features (number of collections, completeness/exclusion criteria, and applied corrections) are summarised in Table 3.

Vasara et al. studied 252 healthy adults in northern Greece [19]. MDP adherence was assessed using a validated 11-item MDP score; potassium exposure was assessed via 24 h urinary potassium excretion and converted to estimated intake using a correction factor (×1.3). Potassium was assessed from a single 24 h urine collection; mean urinary potassium was 65.1 mmol/24 h (corresponding to an estimated intake of 3.3 g/day using the study’s conversion factor), with no gradient across MDP score quartiles. Urinary Na/K also showed no differences across adherence quartiles. In ROBINS-E, overall high risk was driven primarily by high risk in confounding (unadjusted comparisons) and some concerns in participant selection (convenience sampling); outcome measurement was rated low-risk (24 h urine), noting the absence of PABA-based verification and reliance on a single collection (Table 1).

Viroli et al. reported a cross-sectional baseline analysis within a randomised controlled trial in 102 Portuguese university employees [21]. MDP adherence was assessed using a nine-item MDP score; potassium exposure was assessed via a single 24 h urinary excretion. 24 h urinary potassium excretion did not differ between low/moderate- and high-adherence groups (mean 2.6 g/day, i.e., 67.7 mmol/24 h), and tertiles of urinary sodium, potassium, or Na/K were not associated with high adherence after multivariable adjustment. Urinary completeness was screened using sex-specific creatinine-to-body-weight criteria, and urinary potassium values were considered, accounting for ~77% recovery. In ROBINS-E, overall concerns reflected some concerns in confounding, participant selection, and exposure measurement; outcome measurement was rated low-risk (24 h urine), acknowledging that the study had no recovery-marker verification, used creatinine-based screening only, and only a single 24 h collection was available (Table 1).

#### 3.1.3. Longitudinal Evidence

Longitudinal evidence remains limited but provides information on temporal change. Cano-Ibáñez et al. performed a one-year longitudinal analysis within PREDIMED-Plus in 5777 older adults with overweight/obesity and metabolic syndrome [23]. Changes in MDP adherence were assessed using a 17-item energy-restricted questionnaire and categorised by degree of improvement; potassium intake was estimated using a validated 143-item FFQ and expressed as potassium density (mg/1000 kcal). Effect metric: Higher increases in MDP adherence were associated with greater increases in potassium density (mg/1000 kcal), with mean changes of +5.1%, +14.3%, and +25.4% across adherence-change tertiles (≤2/3–5/≥6 points), and multivariable β coefficients of +7.8 and +17.4 mg/1000 kcal for medium and high vs. small changes. In ROBINS-E, the overall high risk was driven mainly by outcome measurement limitations and construct overlap (changes in adherence and potassium inferred from the same FFQ-based dietary shifts), with some additional concerns in confounding and participant selection (Table 1).

#### 3.1.4. Comparative Synthesis Across Observational and Longitudinal Studies

Across questionnaire-/recall-based studies, potassium intake showed consistent positive gradients with increasing MDP adherence. By contrast, biomarker-based analyses relying on single 24 h urinary collections tended to report null or weaker gradients for urinary potassium, and urinary Na/K was generally unchanged across adherence categories where reported. Longitudinal evidence remains limited but directionally consistent with questionnaire-based findings. Notably, higher potassium intake alone does not necessarily indicate improved electrolyte balance; meaningful improvements in Na/K require concurrent reductions in sodium exposure and/or a lower dietary or urinary Na/K ratio.

### 3.2. Evidence from Randomised Controlled Studies

Beyond observational findings, two randomised controlled trials provided interventional evidence on MDP-style dietary patterns and potassium-related outcomes (Table 2). Trial evidence is presented separately because one study was conducted in a chronic kidney disease (CKD) setting with disease-specific dietary constraints [25]. To facilitate comparability, results are summarised using between-group or between-diet differences (Δ) and interpreted in relation to the potassium assessment method (24 h urine vs. dietary recall/spot urine) (Table 2 and Table 3).

Jennings et al. conducted a 12-month, randomised, multicentre intervention within the NU-AGE trial in 1294 Europeans aged 65–79 years (1141 completers) [24]. Participants were randomised to an MDP intervention tailored to older adults versus a habitual diet. Adherence was assessed using the NU-AGE index (0–160), while potassium exposure was assessed via 24 h urinary potassium excretion, with completeness screened using creatinine thresholds (Table 3). Effect metric: The intervention increased 24 h urinary potassium excretion, with a between-group difference of +12.4 mmol/24 h (*p* = 0.01), corresponding to approximately +0.49 g/24; modest sodium reductions were also reported in men. The trial also reported improvements in intermediate vascular outcomes, including reductions in systolic blood pressure and improvements in arterial stiffness. In the RoB 2 appraisal (Table 1), the study was rated as overall some concerns, driven by some concerns in bias due to deviations from intended interventions and missing outcome data, while the randomisation process, outcome measurement, and selection of the reported result were rated low-risk.

Kwon et al. conducted an open-label, randomised two-period crossover pilot trial in 50 Korean adults with stage 3–4 CKD (46 completers), comparing a Mediterranean Proper Optimal Balance (MEDi-POB) diet with a conventional CKD diet [25]. Each dietary phase lasted four weeks and was separated by a four-week washout. MDP adherence, assessed using a validated 14-item Korean MDP score, increased during MEDi-POB. Potassium exposure was assessed using interviewer-administered 24 h dietary recalls and spot urinary potassium (Table 3). Effect metric: Dietary potassium increased modestly during MEDi-POB (Δ = +0.09 g/day; *p* = 0.263) and decreased during the control diet (−0.17 g/day; *p* = 0.072), yielding a borderline between-diet difference (*p* = 0.053), while spot urinary potassium showed no significant change. No significant changes were reported in serum potassium or renal function, alongside reductions in sodium intake during the MEDi-POB phase. In the RoB 2 appraisal (Table 1), the study was rated as overall some concerns, mainly due to some concerns in the randomisation process, deviations from intended interventions, and outcome measurement; missing outcome data and selection of the reported result were rated low-risk.

Taken together, these trials indicate that MDP-style interventions can increase MDP adherence and, in some settings, translate into measurable increases in potassium exposure. However, potassium-related changes were not uniform across trials and differed by population context and potassium assessment method (24 h urine vs. recall/spot urine) (Table 2 and Table 3).

## 4. Discussion

**Figure 2:**
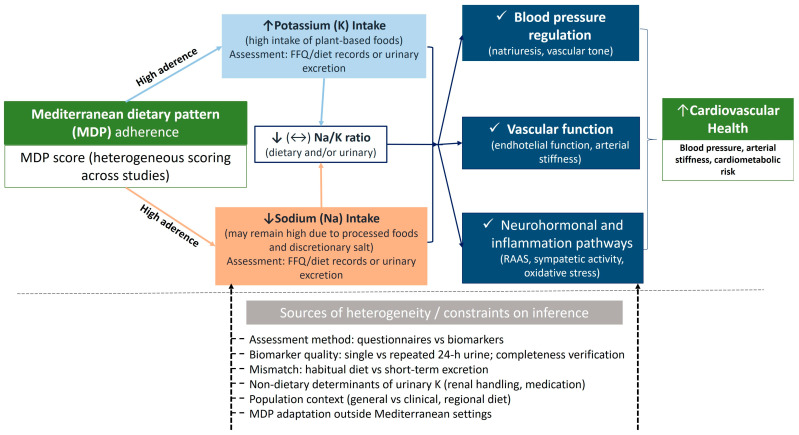
Conceptual framework linking Mediterranean dietary pattern (MDP) adherence, potassium exposure, sodium exposure and sodium-to-potassium (Na/K) balance, and cardiometabolic outcomes. The diagram summarises hypothesised pathways through which higher MDP adherence may relate to cardiometabolic health. The framework is illustrative and hypothesis-generating and does not imply causal direction. Higher adherence is expected to increase potassium intake (K) via greater consumption of potassium-rich foods (e.g., fruit, vegetables, legumes, nuts, whole grains), while sodium exposure (Na) may decrease, remain unchanged, or remain high depending on discretionary salt use, processed foods, and condiment-based seasoning. The Na/K ratio represents an integrative marker of electrolyte balance and may better capture cardiometabolic risk than either electrolyte alone. Downstream outcomes (e.g., blood pressure and vascular function) are shown as potential consequences of these dietary exposures. The framework also highlights key sources of heterogeneity and measurement considerations relevant to interpretation, including differences in MDP score definitions, potassium assessment method (dietary instruments vs. urinary biomarkers), and the need for repeated, completeness-verified 24 h urine collections to approximate usual exposure. Abbreviations: MDP, Mediterranean dietary pattern; K, potassium; Na, sodium; Na/K, sodium-to-potassium ratio; RAAS, renin–angiotensin–aldosterone system.

This narrative review synthesises epidemiological and interventional evidence on the association between adherence to the MDP and dietary potassium intake. Overall, higher MDP adherence was generally associated with greater potassium intake, consistent with the plant-forward composition of the pattern. However, the strength and consistency of this relationship varied according to how MDP adherence was defined and quantified, how potassium exposure was assessed (self-reported intake versus urinary biomarkers), and population-specific dietary practices (Figure 2).

Given the predominantly observational and often cross-sectional evidence base, findings should be interpreted as associations rather than causal effects. Mechanistic pathways are discussed as biologically plausible hypotheses and mediation cannot be inferred without formal mediation analyses.

Interventional evidence remains limited, but the available trials included in this review suggest that MDP-style interventions can improve adherence and, in some settings, increase potassium exposure. In the NU-AGE multicentre trial, improved MDP adherence was accompanied by higher 24 h urinary potassium excretion and favourable changes in intermediate vascular outcomes within this single large trial [24]. In the trial, urinary potassium was derived from a single 24 h collection with creatinine-based completeness screening, but without repeat collections or recovery-marker verification, which may still limit inference on usual exposure. In the CKD crossover trial, potassium was assessed primarily via dietary intake alongside spot urine measures, which are less suited to quantifying usual 24 h excretion and further constrain comparability with observational biomarker studies [25]. Recent exploratory evidence not eligible for inclusion (e.g., the Keto–Salt Pilot Study) is directionally consistent, reporting blood pressure reductions alongside only modest, non-significant changes in urinary potassium derived from a single 24 h collection. However, inference is constrained by small sample size, short duration, and limited biomarker sampling [30].

Observational evidence is more extensive but also more heterogeneous, and the risk-of-bias appraisal suggests that methodological differences contribute materially to variability in effect estimates. Most studies report positive associations between MDP adherence and potassium intake, which is biologically coherent given the centrality of potassium-rich plant foods within this pattern. Nevertheless, the magnitude of association may be influenced by shared measurement structures when adherence scores and nutrient estimates derive from the same self-reported instrument. Thus, questionnaire-based concordance should be interpreted primarily as pattern-consistent rather than nutrient-specific evidence. Contextual observational findings from specific populations not included in this review (e.g., older adults at elevated cognitive risk and pregnancy cohorts) further illustrate both coherence and limits of inference: higher MDP adherence may co-occur with higher potassium exposure, yet sodium exposure can remain high and Na/K may not improve, and cross-sectional designs with limited biochemical validation constrain generalisability and causal interpretation [31,32].

### 4.1. Heterogeneity by Assessment Method and Exposure Timeframe

We interpret consistency and robustness in light of study design (cross-sectional/longitudinal/randomised controlled trial) and potassium assessment method (questionnaire vs. urinary biomarkers, with attention to repeat sampling and recovery-marker verification, alongside creatinine-based screening). Differences between questionnaire-based and biomarker-based findings are best interpreted through the lens of what each method measures. Dietary instruments (FFQs/recalls) are designed to capture habitual intake and therefore align conceptually with MDP adherence scoring. However, they remain vulnerable to reporting biases (including recall and social desirability bias), portion-size inaccuracy, and variability in food-composition databases, which may inflate associations or introduce exposure misclassification. Diet history methods fall within the same construct, as they aim to capture habitual dietary patterns and therefore map closely onto the food-based structure underlying MDP scores. In contrast, 24 h urinary potassium provides a more objective index of recent exposure, yet its validity as a marker of usual intake depends critically on repeated collections and verified completeness. When urinary potassium is based on a single 24 h collection, substantial within-person day-to-day variability is expected to introduce regression dilution and attenuate associations with habitual diet patterns. Accordingly, weaker or null associations in biomarker-based studies are plausibly driven by reliance on single 24 h collections without recovery-marker verification and by substantial within-person day-to-day variability (regression dilution), even when creatinine-based screening is applied to exclude clearly incomplete samples. Taken together, the limited number of studies and the heterogeneity in exposure assessment (predominantly self-report, with sparse urinary biomarker data) do not allow formal triangulation of evidence. Rather, the observed pattern highlights the need for future studies explicitly designed to integrate dietary instruments, repeated validated 24 h urine collections, and intervention evidence.

Biomarker-based analyses are also often conducted in smaller and more selected samples, which can further limit precision and generalizability. Accordingly, cautious interpretation is warranted when urinary collections are limited in number and/or completeness is not verified. Overall, this inherent mismatch, habitual diet versus short-term excretion, offers a coherent explanation for why questionnaire-based studies often report clearer gradients than urinary analyses based on single collections.

### 4.2. Sodium–Potassium Balance as the Mechanistically Relevant Exposure

Potassium should be interpreted alongside sodium, because vascular risk and blood pressure regulation are more closely related to electrolyte balance than to either nutrient in isolation. Within the included evidence base, Na/K was reported in only a small subset of analyses (diet-based and/or urinary), and where assessed it was unchanged in urinary analyses where reported, whereas diet-based estimates suggested a more favourable dietary Na/K profile in some cohorts (driven primarily by higher potassium intake with relatively stable sodium exposure, consistent with the observation that Na/K may remain unchanged when sodium exposure does not concurrently decrease). Na/K is, therefore, a physiologically coherent and clinically relevant integrative metric, capturing the combined haemodynamic and vascular consequences of dietary exposure. Higher potassium intake may promote natriuresis and support vascular function, thereby attenuating sodium-related volume expansion and vascular dysfunction [9]. However, persistently high sodium exposure may offset these potential benefits, making the net balance the more informative target for interpretation and prevention strategies [33,34,35,36]. Importantly, the interpretability of Na/K is strengthened when estimated from robust exposure measures (e.g., repeated 24 h urinary collections), whereas single or less reliable measures may attenuate associations.

This perspective is particularly useful for interpreting heterogeneity across settings, since MDP adherence may increase potassium intake while sodium intake remains unchanged, or even higher, in contexts characterised by substantial discretionary salt use or sodium-rich condiments. Accordingly, an apparent lack of improvement in Na/K should not be interpreted as an absence of potassium-related dietary change, but rather as evidence that concurrent sodium patterns may blunt the expected shift in balance. Evidence from large cohorts supports the clinical relevance of this construct: higher urinary Na/K has been associated with higher cardiovascular risk when assessed using repeated 24 h urine collections, and higher dietary Na/K has been linked to stroke, cardiovascular disease and all-cause mortality in prospective cohort data [33,37].

From a reporting and synthesis standpoint, future studies should routinely present sodium, potassium and Na/K together (dietary and/or urinary) and interpret potassium-related associations within the broader sodium–potassium context. This is equally relevant for interventions, where improvements in vascular outcomes may depend not only on increasing potassium intake but on achieving a meaningful improvement in overall sodium–potassium balance. Finally, population-level strategies specifically designed to improve sodium–potassium balance, such as potassium-enriched, reduced-sodium salt substitutes, have demonstrated cardiometabolic benefits in large trials [38,39,40]. However, these approaches are conceptually distinct from dietary patterns such as the MDP and lie outside the primary scope of the present review.

### 4.3. Contemporary Adaptations of the MDP in Non-Mediterranean Settings

Contemporary “MDP-like” adaptations implemented in non-Mediterranean regions are often necessary to maximise feasibility and acceptability, but they should not be assumed to reproduce the mineral profile of traditional Mediterranean diets. Figure 1 illustrates the wide geographic spread of the evidence base, which is likely to contribute to heterogeneity given setting-specific differences in food availability, processing, and culinary practices relevant to potassium and sodium exposure. Cultural adaptation frameworks emphasise preserving core pattern features while recognising that local food environments, the availability of potassium-rich foods, and food processing and preparation practices can materially alter mineral exposures [41,42]. In East Asian settings, for example, a Mediterranean-aligned pattern may increase potassium-rich foods (e.g., vegetables and fruits, fish, and dairy), which are major contributors to potassium intake in Japanese adults [43]. However, sodium exposure may remain comparatively high because it is frequently driven by discretionary salt use and condiment-based seasoning; detailed analyses of Japanese diets indicate that seasonings and other culturally specific sources account for a substantial share of total sodium intake [43,44]. As a result, MDP adherence may be associated with higher potassium intake without proportional reductions in sodium exposure, leading to smaller improvements in overall electrolyte balance (Na/K) than might be expected from Mediterranean cohorts [43]. These considerations support reporting potassium alongside sodium (and, where available, Na/K) when evaluating culturally adapted MDP scores and caution against assuming equivalence in mineral composition across regions despite similar food-group-based adherence metrics [41,42]. Cross-cohort comparability is further constrained by heterogeneity in adherence scoring, ranging from brief nine-item indices to more extensive multi-component scores, resulting in partially non-overlapping operational definitions of the MDP construct (i.e., differences in the food components included, cut-offs, and scoring rules used to define “high adherence”) across studies and complicating cross-cohort interpretation.

### 4.4. Potassium as Mediator Versus Marker of Diet Quality: Implications for Inference

From an aetiological standpoint, potassium is a biologically plausible contributor to vascular benefits, yet it is also strongly correlated with the broader nutritional profile of an MDP (fibre, polyphenols, magnesium and lower ultra-processed food intake). This clustering complicates causal attribution in observational data and cautions against interpreting potassium as the sole driver of MDP-related effects. Disentangling potassium-specific effects from correlated dietary components will require analytic approaches capable of evaluating multiple, simultaneous pathways that incorporate both nutrient-based and food-based characteristics of the MDP. Accordingly, potassium may be best conceptualised as a candidate mediator within a broader pattern-level exposure, rather than an isolated causal factor. Notably, observational data, particularly cross-sectional designs, cannot establish mediation and should be interpreted as hypothesis-generating.

To advance beyond plausibility, future studies should adopt mediation-oriented analytic frameworks (e.g., counterfactual mediation approaches or structural models that accommodate correlated exposures) to quantify the extent to which changes in potassium and Na/K account for improvements in blood pressure, vascular function and related outcomes. Such analyses require robust exposure assessment, particularly repeated/validated biomarkers where feasible, and careful control of confounding and co-exposures.

### 4.5. Risk-of-Bias Considerations

The risk-of-bias appraisal (Table 1) indicates that most cross-sectional studies were judged at high risk of bias, and the available longitudinal evidence was also rated high risk of bias, whereas randomised trials were generally assessed as having some concerns.

Overall, judgements were primarily driven by the exposure/outcome assessment structure, particularly the degree of independence between MDP adherence scoring and potassium estimation (common-method structure/construct overlap), as well as by the quality of potassium assessment (self-report vs. urinary biomarkers). Residual confounding generally contributed to some concerns rather than high-risk judgments.

These limitations, together with frequent reliance on convenience sampling and single 24 h urinary collections without repeat sampling and recovery-marker verification in biomarker-based studies, are expected to increase misclassification of usual exposure and should be interpreted with caution regarding causal inference. Specifically, common-method structure may inflate concordance in questionnaire-based studies, whereas within-person variability and imprecise capture of usual exposure due to single collections and substantial day-to-day variability may attenuate associations in urinary biomarker analyses.

Additional review-level limitations should also be considered. Given the narrative scope and single-database retrieval, incomplete capture of the evidence base and publication bias cannot be excluded. Because the search relied primarily on title/abstract indexing within a single bibliographic database, relevant studies may have been missed. In addition, the included evidence base is characterised by conceptual heterogeneity in MDP score definitions (differences in components, cut-offs, and scoring rules), which limits cross-study comparability and may contribute to exposure misclassification and between-study heterogeneity. Finally, screening, study selection, and data extraction were undertaken by a single reviewer, which may have increased the risk of selection or extraction errors and subjective interpretation.

### 4.6. Methodological Priorities and Practical Implementation

To enhance cross-study comparability, future research should converge on a core set of MDP components with standardised food-group definitions and scoring rules, and transparently apply cross-walk/standardised procedures when alternative indices or locally adapted scores are used. Given feasibility constraints in large cohorts, a scalable strategy is a two-stage design: (i) standardised dietary assessment and MDP scoring in the full sample, and (ii) repeated 24 h urine collections (≥2, ideally 3 across different time points/seasons) in a representative subsample, supported by standard operating procedures and completeness checks (e.g., PABA where feasible, or creatinine-/volume-based plausibility criteria). Subsample biomarker data should then inform measurement-error correction (e.g., regression calibration or mixed-effects models) to estimate usual potassium exposure and limit regression dilution. Where possible, potassium should be reported alongside sodium, also considering the Na/K ratio to facilitate interpretation and synthesis across settings. Finally, integrating dietary metrics, repeated biomarkers and intervention evidence offers a pragmatic triangulation framework to strengthen confidence in the robustness and plausibility of the observed associations regarding potassium within the broader MDP exposure profile. Under a triangulation framework, convergence of associations across (i) questionnaire-derived habitual intake, (ii) completeness-verified repeated 24 h urinary excretion, and (iii) intervention-induced changes in intermediate vascular outcomes would support stronger causal interpretation, while discordance would highlight method-specific error or context-dependent effects.

## 5. Conclusions

Overall, higher adherence to the MDP was generally associated with higher potassium intake, although estimates varied across MDP score definitions and potassium assessment methods (dietary instruments vs. urinary biomarkers). These patterns are consistent with greater consumption of potassium-rich foods and may contribute to a more favourable Na/K balance, contingent on concurrent sodium exposure. Biomarker-based findings were often weaker, likely reflecting methodological constraints, including limited repeat sampling and lack of recovery-marker verification (with creatinine-based screening used in some studies), and the conceptual mismatch between habitual diet and short-term urinary excretion. While biological plausibility and trial evidence support a contributory role of potassium within the broader cardiometabolic profile of the MDP, its independent contribution cannot yet be quantified reliably. Evidence from CKD trials should be interpreted as setting-specific and is not directly generalisable to free-living populations without renal impairment. Findings should also be interpreted in light of the narrative scope, potential incomplete retrieval and publication bias, and conceptual heterogeneity across MDP scores. Public health strategies that promote MDP adherence, alongside reductions in sodium exposure and ultra-processed foods and improved access to potassium-rich foods, represent a pragmatic approach to improving overall diet quality and supporting NCD prevention [45,46].

## Figures and Tables

**Table 1:** Assessment of risk of bias for studies included in the review.

Cross-Sectional Design								
Study Name, Year (Ref.)	Confounding	Selection of Participants in the Study	Measurement of Exposure	Post-Exposure Interventions	Missing Data	Measurement of the Outcome	Selection of the Reported Result	Overall Risk of Bias
Serra-Majem et al. (2009) [16]	Some concerns	Low risk	Some concerns	N/A	Low risk	High risk	Low risk	High risk
Feart et al. (2012) [17]	High risk	Low risk	Some concerns	N/A	Low risk	Some concerns	Low risk	High risk
Kanauchi et al. (2016) [18]	Some concerns	Some concerns	Some concerns	N/A	Low risk	High risk	Low risk	High risk
Malavolti et al. (2021) [20]	Some concerns	Some concerns	Some concerns	N/A	Low risk	High risk	Low risk	High risk
Rumawas et al. (2009) [22]	Some concerns	Some concerns	Some concerns	N/A	Low risk	High risk	Low risk	High risk
Vasara et al. (2017) [19]	High risk	Some concerns	Some concerns	N/A	Low risk	Low risk	Low risk	High risk
Viroli et al. (2021) [21]	Some concerns	Some concerns	Some concerns	N/A	Low risk	Low risk	Low risk	Some concerns
**Longitudinal Design**								
Cano-Ibáñez et al. (2020) [23]	Some concerns	Some concerns	Some concerns	Uncertain	Low risk	High risk	Low risk	High risk
**Randomised Controlled Trial**								
**Study name, year [Ref.]**	**Bias arising from the randomisation process**	**Bias due to deviations from intended interventions**		**Bias due to missing outcome data**	**Bias in the measurement of the outcome**		**Bias in the selection of the reported result**	**Overall risk of bias**
Jennings et al. (2019) [24]	Low risk	Some concerns		Some concerns	Low risk		Low risk	Some concerns
Kwon et al. (2024) [25]	Some concerns	Some concerns		Low risk	Some concerns		Low risk	Some concerns

Higher overall risk-of-bias judgements in observational studies were mainly driven by (i) common-method structure/construct overlap when MDP adherence and potassium were derived from the same self-reported instrument, and (ii) exposure/outcome measurement limitations (e.g., single FFQ/24 h recall-derived estimates without biomarker corroboration). Residual confounding, reflecting incomplete adjustment for pre-specified critical confounders, generally contributed to some concerns rather than high-risk judgments. For urinary biomarkers, 24 h collections were generally rated low risk, whereas spot urine potassium was considered more prone to misclassification and contributed to a less favourable judgement.

**Table 2:** Characteristics of the studies reporting the relationship between Mediterranean dietary pattern (MDP) and potassium intake in adult populations.

Cross-Sectional Design						
Potassium Intake Assessment by Questionnaire						
First Author (Year) [Ref.]	Country	Participants (*n*)	MDP Adherence Assessment Method	Potassium Intake Assessment	Effect Metric (Potassium) and Adjustment	
Serra-Majem et al. (2009) [16]	Europe (Spain)	17,197	A posteriori MDP derived from FFQ (136 items)	Semi-quantitative FFQ (136 food items)	Dietary K (g/day) Q1 → Q5 3.7 → 6.1; Western Q1 → Q5 5.4 → 4.2; Adj: energy (residual method)	
Feart et al. (2012) [17]	Europe (France)	1595	FFQ (148 items, 40 food categories)	24 h dietary recall with portion assessment	Dietary K (g/day) MDP 0–3/4–5/6–9: men 2.9 → 3.0 → 3.1 (*p* = 0.04), women 2.4 → 2.5 → 2.7 (*p* < 0.0001); Adj: none; Analysis: ANOVA (sex-stratified)	
Kanauchi et al. (2016) [18]	Asia (Japan)	1048	BDHQ (58 items)	BDHQ (58 items)	Dietary K (g/day) MDP 0–4/5–7/8–13: 2.5 → 3.0 → 3.5; Adj: none; Analysis: descriptive.	
Malavolti et al. (2021) [20]	Europe (Italy)	719	Semi-quantitative FFQ (EPIC—188 food items)	Semi-quantitative FFQ (EPIC—188 food items)	Dietary K (g/day) IMI < 4 vs. ≥4: 3.1 → 3.6; MIND < 7.5 vs. ≥7.5: 3.1 → 3.6 (both *p* < 0.001); Adj: none; Analysis: *t*-test. Dietary Na/K improved (Na ↔, K ↑)	
Rumawas et al. (2009) [22]	North America (USA)	3021	MDP-Style Score (0–100) derived from FFQ (126 items—13 food groups)	Semi-quantitative FFQ (126 items)	Dietary K (g/day) Q1 → Q5: 2.4 → 3.3 (P-trend < 0.001); Adj: age, sex, energy.	
**Potassium Intake Assessment by Urine Collection**						
Vasara et al. (2017) [19]	Europe (Greece)	252	11-item Mediterranean diet score	24 h urine collection	U-K (mmol/24 h) quartiles: ns (K *p* = 0.735); mean 65.1 mmol/24 h; Adj: none; Analysis: ANOVA; QC: creatinine/time (no PABA); urinary Na/K: ns.	
Viroli et al. (2021) [21]	Europe (Portugal)	102	9-item MDP score derived from FFQ (82 items)	24 h urine collection	U-K (mmol/24 h) low/mod vs. high adherence: ns; mean 67.7 mmol/24 h; Adj: age, BMI, energy, Edu, HTN, PA; QC: creatinine/weight; urinary Na/K: ns.	
**Longitudinal Design**						
Cano-Ibáñez et al. (2020) [23]	Europe (Spain)	5777	Energy-restricted MDP adherence questionnaire (17 items)	Semi-quantitative FFQ (143 food items)	K density (mg/1000 kcal) tertiles (≤2/3–5/≥6): %Δ +5.1/+14.3/+25.4; β +7.8/+17.4 mg/1000 kcal; Adj: multivariable.	
**Randomised Controlled Trial**						
**First author (year) [** **R** **ef** **.** **]**	**Country**	**Participants (n)**	**MDP adherence assessment method**	**Potassium intake** **assessment**	**Comparison**	**Effect Metric (potassium) and Adjustment**
Jennings et al. (2019) [24]	Europe (Italy, UK, The Netherlands, Poland, France)	1294 [1141 completers] Older adults	7-day food diaries (NU-AGE index: 16 dietary components)	24 h urine collection	RCT: MDP vs. habitual diet (12-month intervention)	U-K (mmol/24 h) BG Δ +12.4 (*p* = 0.01) (~+0.49 g/24 h); Adj: ANCOVA; QC: creatinine-screened (single 24 h).
Kwon et al. (2024) [25]	Asia (Korea)	50 [46 completers] stage 3–4 CKD	FFQ (14 items)	24 h dietary recall and spot urine	RCT: Korean-style MEDi-POB diet vs. conventional CKD diet (4-week intervention + 4-week washout)	Dietary K (g/day) within-diet Δ +0.09 vs. −0.17; between-diet Δ −0.26 (*p* = 0.053); Adj: LMM (sequence, period); spot U-K: ns.

Adj = adjustment; ANCOVA = analysis of covariance; ANOVA = analysis of variance; BDHQ = Brief-type Self-Administered Diet History Questionnaire; BG Δ = between-group difference; CKD = chronic kidney disease; Dietary K = dietary potassium intake (g/day), estimated from FFQ/recall; DM = diabetes mellitus; Edu = educational level; FFQ = food frequency questionnaire; HTN = hypertension; IMI = Italian Mediterranean Index; K density = potassium density (mg/1000 kcal); LMM = linear mixed model; MDP = Mediterranean dietary pattern; MEDi-POB = Mediterranean Proper Optimal Balance; ns = not significant; MIND = Mediterranean–DASH Intervention for Neurodegenerative Delay; NU-AGE = New Dietary Strategies Addressing the Specific Needs of Elderly Population for Healthy Aging in Europe; PA = physical activity; Q1–Q5 = quintiles; QC = urine quality/completeness checks; RCT = randomised controlled trial; U-K = 24 h urinary potassium excretion (mmol/24 h).

**Table 3:** Urinary potassium assessment characteristics in studies included in the review.

First Author (Year) [Ref.]	Design/Setting	Urine Measure	Number of Collections	Completeness/Exclusion Criteria	Correction Applied	Notes
Vasara et al. (2017) [19]	Cross-sectional	24 h	1	creatinine-based exclusions	×1.3 (conversion to intake)	convenience sampling; exclusions by creatinine thresholds; no repeat collections; unadjusted
Viroli et al. (2021) [21]	Cross-sectional	24 h	1	no recovery marker; creatinine-based screening only	~77% recovery adjustment	convenience-based employees; analysis level NR
Jennings et al. (2019) [24]	Randomised controlled trial	24 h	1	creatinine thresholds	None (excretion reported)	multicentre; inter-site variability possible; analysis level NR
Kwon et al. (2024) [25]	Randomised controlled trial	Spot	1	Not applicable	None reported	CKD stage 3–4; spot K not proxy for habitual intake; analysis level NR

CKD: chronic kidney disease; NR: not reported.

## Data Availability

No new data were created or analyzed in this study. Data sharing is not applicable to this article.

## Abbreviations

The following abbreviations are used in this manuscript:

BDHQ	Brief-type Self-Administered Diet History Questionnaire
CKD	Chronic Kidney Disease
EPIC	European Prospective Investigation into Cancer
FFQ	Food Frequency Questionnaire
IMI	Italian Mediterranean Index
MDP	Mediterranean dietary pattern
MEDi-POB	Mediterranean Proper Optimal Balance
MIND	Mediterranean–DASH Intervention for Neurodegenerative Delay
NCDs	Non-Communicable Diseases
NU-AGE	New Dietary Strategies Addressing the Specific Needs of Elderly Population for Healthy Aging in Europe
PABA	Para-Aminobenzoic Acid
PICOS	Population, Intervention/Exposure, Comparison, Outcomes, Study Design
PREDIMED	Prevención con Dieta Mediterránea
